# Smart Coating of Carbon Steel Using Polystyrene Clay Nanocomposites Loaded with Cerium and Silanol Inhibitors: Characterization and Electrochemical Study

**DOI:** 10.3390/polym16223196

**Published:** 2024-11-17

**Authors:** Layla A. Al Juhaiman, Mona A. Al Jufareen, Saeed M. Al-Zahrani, Ubair Abdus Samad, Tahani S. Al-Garni

**Affiliations:** 1 Chemistry Department, Science College, King Saud University, Riyadh 145111, Saudi Arabia; mjufareen@gmail.com (M.A.A.J.); tahanis@ksu.edu.sa (T.S.A.-G.); 2 Chemical Engineering Department, Engineering College, King Saud University, Riyadh 11421, Saudi Arabia; szahrani@ksu.edu.sa; 3 Center of Excellence for Research in Engineering Materials (CEREM), King Saud University, Riyadh 11421, Saudi Arabia; uabdussamad@ksu.edu.sa

**Keywords:** carbon steel, Khulays clay, polystyrene clay nanocomposites, nanoindentation tests, microcapsules, electrochemical impedance spectroscopy, smart coating

## Abstract

Local Khulays clay was modified to prepare polystyrene clay nanocomposite (PCN) coatings on carbon steel. The PCN coatings were added to microcapsules (MCs) loaded with the corrosion inhibitor PCN(MC). The microcapsules were prepared by the encapsulation of rare-earth metal Ce^+3^ ions and isobutyl silanol into polystyrene via the double emulsion solvent evaporation (DESE) technique. From characterization techniques, Fourier-transform infrared spectroscopy (FT-IR), X-ray diffraction (XRD), transmission electron microscopy (TEM), and scanning electron microscopy (SEM) with EDX. SEM and FT-IR confirmed the success of the preparation of the PCN(MC). Nanoindentation tests were performed on the thin-film samples. A significant reduction in both the hardness and the reduced modulus was observed for the PCN film compared to the PS film. Electrochemical impedance spectroscopy (EIS) and electrochemical frequency modulation (EFM) all showed an enhanced protection efficiency (%PE) of 3% PCN(MC) over 3% PCN at high temperatures and at different times. The smart coatings were proven by applying the thermal and the mechanical triggers for the 3% PCN(MC) coating. The mechanism of the release of inhibitors was discussed. The self-healing properties of 3% PCN(MC) were evaluated. The enhanced properties of the developed PCN(MC) coatings make them attractive for potential applications in the oil and other industries.

## 1. Introduction

One of the most significant current issues in industry is metal corrosion. Corrosion is a natural electrochemical phenomenon with progressive detrimental effects on the integrity of materials and huge economic losses for various industries. The global cost of corrosion is estimated to be USD 2.5 trillion [1]. Controlling corrosion is one of the biggest challenges in industry. Therefore, protecting steel and its alloys from corrosion is important for minimizing economic losses, improving cost reduction, reducing malfunction dangers, and achieving acceptable performance [1]. There are five primary methods for corrosion control: material selection, design, inhibitor use, cathodic protection, and coating [1,2]. Every method has its benefits. In our study, we aim to protect steel and steel bodies against corrosion. As researchers and scientists, we aim in this study to find the best coating for steel and steel bodies like carbon steel in corrosive environments like sea water. However, the coating resistance will not last for a long time and it may be penetrated, resulting in an internal pathway through which the corrosive substances pass to the metal surface, causing oxidation of the metal [3,4,5]. Therefore, a new generation of high-performance protective coatings that can provide long-term protection is required. Organic coatings, which act as a physical barrier for isolating metal surfaces from destructive conditions, are the most common approaches for the corrosion protection of metallic structures. However, organic coatings cannot provide long-term corrosion protection because of their brittle structure and hydrolytic degradation during exposure to destructive conditions. Moreover, pores were created inside the polymer matrix during the curing process because of the evaporation of organic solvents and external environmental stresses. These micro-pores provide a path for the diffusion of corrosive agents (such as water molecules and chloride ions) through the coatings to the metal substrate [2]. To increase the efficiency of organic polymer coatings, they are intercalated with layered structures like clay [3,4,5,6]. Polymer clay nanocomposites are multiphase systems in which the nanoparticles with at least one dimension in a nanoscale system are distributed in the polymer matrix [4]. Generally, there are three different methods for synthesis of polymer clay nanocomposites: solution-blending method, melt-blending method, and in situ polymerization method [3,4]. In the present study, we applied the solution-blending method to prepare polystyrene organoclay nanocomposites (PCNs) [5].

Smart coatings are functional systems whose characteristics may be altered in a controlled manner in response to a stimulus generated by an intrinsic or extrinsic event [7,8,9,10,11]. Smart coatings first require a trigger that permits them to respond to the environment. Usually, the trigger mechanism is chemical, mechanical, or thermal [7]. Next, a repair mechanism commonly includes a way to hold the repair agent (inhibitor) within the coating and a means of releasing it [7]. It includes a protective mechanism that depends on the adsorption of water molecules from the environment [7]. The other one is called encapsulation where a proper self-healing system should remain steady under numerous environmental situations, be easily encapsulated, and respond rapidly to the repair process once triggered [7]. The capsule is a reservoir for inhibitors and healing agents. Various triggering mechanisms can be used to initiate a response in coating systems. These triggers may be activated autonomously or externally. The decision between these two relies on the application and the degree of outer oversight that is required. Triggering is based on the pH and mechanical triggering, thermal triggering, and ion-exchange processes [7,8]. The most common methods and technologies used for the preparation of smart coating are chemical conversion coatings, nanocapsule- and microcapsule-based polymer coatings, layer-by-layer (LbL) coatings, self-assembly coatings, molecular deposition coatings, shape memory (SM) coatings, and self-healing coatings for carbon nanotubes and clay nanotubes [7,8].

Several types of smart systems have been used. Hoseinzadeh and Javadpour [9] used halloysite nanotubes (HNTs) as carriers for the inhibitor 3,4′-dihydro-3-[2′-mercaptothiazolidine]indol-2-one (DMI). Based on their results, the 2.5 wt.% DMI-loaded HNT samples had the highest and best corrosion resistance. On the other hand, Takeshi Matsuda et al. [10] used cerium nitrate as a corrosion inhibitor encapsulated in urea-formaldehyde (UF) by a water-in-oil emulsion. The results of the electrochemical analysis showed that MCs with the smallest size provided more corrosion protection than the largest ones. Moreover, Koh and Park [11] synthesized polyurethane microcapsules (PUMCs) by using the interfacial polymerization method. A scratched coating surface was used to investigate the healing abilities of the prepared coatings.

Extending previous studies [5,12,13,14], the aim of this work was to prepare PCN then apply the method of Cotting et al. [15] to prepare smart coatings using microcapsules impregnated with inhibitors. We used Khulays raw clay (RC) from Saudi Arabia. We prepared polystyrene clay nanocomposite coatings and doped these nanocomposites with corrosion inhibitors like cerium and silane to increase the coating efficiency. Characterization methods such as X-ray diffraction (XRD), Fourier-transform infrared spectroscopy (FT-IR), scanning electron microscopy (SEM), transmission electron microscopy (TEM), and nanoindentation test were applied. Microhardness testing and nanoindentation are the standard methods for determining the hardness of materials. Nanoindentation was applied in the present work and has the added benefit of providing the elastic modulus. We also evaluated the anticorrosive properties of these polymer nanocomposites using electrochemical methods (electrochemical impedance spectroscopy and electrochemical frequency modulation). A thermal trigger and mechanical trigger were followed by electrochemical impedance spectroscopy (EIS). Comparing the results of EIS data at different temperatures and longer immersion times for the coating with and without the encapsulated inhibitor helped us understand the release mechanism of the inhibitors and the mechanism of smart coating.

## 2. Materials and Methods

### 2.1. Materials

The metal to be protected is carbon steel (C-steel), provided by ODS Co., Schleswig-Holstein, Germany. The main constituents are 98.648% iron, 0.46% carbon, and 0.60% manganese with other alloying elements as described earlier [12].

The local Khulays raw clay (RC) was collected by a certified geologist from the Khulays region, Saudi Arabia. It is a bentonite clay mineral [13]. The RC contains montmorillonite (35.22%), kaolinite (13.33%), mica (22.80%), quartz (8.57%), feldspar (6.66%), ilmenite (5.71), dolomite (3.81%), and gypsum (3.81%).

Different types of materials were used in this study. Polystyrene (PS) was used as the matrix for PCN and MC Shell. The inhibitors used were Ce(NO_3_)_3_·6H_2_O and isobutyl trimethoxy silane (IBTMS) 97% as will be discussed in the preparation method.

### 2.2. Preparation of Polystyrene/Organoclay Nanocomposites

We prepared a polymer clay nanocomposite (PCN) as a protective coating for C-steel according to the procedure explained earlier [5,12,14]. The raw clay (RC) was washed with distilled water then treated with NaCl to prepare the NaC. The cationic surfactant cetyl pyridinium chloride (CPC) was used in the ion exchange treatment to convert the NaC into the organoclay form (OC) so that it is compatable with PS. The resulting organoclay (OC) was separated by centrifugation and washed with distilled water then dried in the oven as explained in other studies [5,12].

Applying the solution-blending method, a certain weight of organoclay (OC) (0.02 g and 0.06 g) was added to 10 mL of toluene solvent in a 50 mL flask to prepare 1% and 3% PCN. The mixture was magnetically stirred (350 rpm) at room temperature for 24 h. Subsequentially, 2 g of polystyrene (matrix) was added to each suspension under the same condition for 24 h. Then, the nanocomposite was sonicated for 10 min.

### 2.3. Preparation of Microcapsules Loaded with Corrosion Inhibitors

We started the present study after the Corona pandemic when buying new chemicals from abroad was so difficult and time-consuming (8–10 months). The choice of cerium corrosion inhibitor and silane was based on a study of Cotting et al. [15]. They used epoxy resin and octylsilanol (MW 276.48). We tried the available silane, which was IBTMS (MW 178.30), and it worked so well. The microcapsules loaded with corrosion inhibitors were prepared by the double emulsion solvent evaporation method [15]. This method includes three main steps and labels with (W1), (W1/O), and (W1/O/W2).

The first step was W1 preparation in 100 mL flask. A 50/50% equal volume of distilled water and 96% ethanol was prepared. Then, 5000 ppm of Ce(NO_3_)_3_·6H_2_O was added in addition to 4% of isobutyl trimethoxy silane (pH adjustment at 5 by NaOH). W1 was magnetically stirred for 24 h at room temperature to complete the hydrolysis process ending with condensation of the solution.

The second step included W1/O preparation, in which 2 g of polystyrene was dissolved in 10 mL dichloromethane (DCM), followed by the addition of 0.5 g of CPC surfactant. Then, 1 mL of the corrosion inhibitor solution W1 was added and sonicated for two minutes. In the third step, a flask with an amount of 30 mL of 1% PVA under magnetic stirring (200 rpm) and then the W1/O was added slowly, and a large amount of 0.3% PVA (100 mL) was the final addition. The solution was magnetically stirred (200 rpm) for 24 h at room temperature to evaporate the solvent. The polystyrene microcapsules (MCs) were filtered using a Buchner funnel and then washed several times with deionized water to remove PVA and non-encapsulated inhibitors. The microcapsules were gently collected in a crucible and placed in a CaCl_2_ desiccator.

### 2.4. Pretreatment of C-Steel Electrodes and Casting

In this study, the C-steel rods were used as a working electrode. The surface area of each rod was 4.75 cm^2^, as measured by a Mitutoyo gauging tool (Kawasaki, Kanagawa, Japan). The C-steel electrodes were polished using abrasive papers of grades 60 and 400 using a polishing machine (Metaserv 2000, Buehler, Coventry, UK). The purpose of polishing the surface is to remove any deposits and impurities and provide a slight roughness that increases the adhesion of the coating. Subsequently, the C-steel surface was washed with distilled water and immersed in acetone under ultrasonication for 2 min. In each experiment, three C-steel electrodes were prepared. Next, the C-steel electrodes were left to dry. For casting, the PCN solution was applied dropwise to the C-steel surface as the first layer and left to dry in atmospheric air for 4 h. Similarly, the second layer was applied and then dried in a furnace (Carbolite, Neuhausen, Germany) for 24 h at 60 °C. Then, the coating thickness was determined using a coating thickness gauge (Elcometer 456, Madison, UK). The thickness was maintained in the range of 100 ± 10 µm. For the nanocomposite coating impregnated with the microcapsules (MCs), a few drops of the suspended MCs in isopropyl alcohol were introduced onto the clean electrode surface and left at room temperature until the alcohol evaporated. Then, the previous steps were used for the PCN casting as described previously.

### 2.5. Characterization Methods

Various characterization methods were used to demonstrate successful PCN preparation and ensure the effect of corrosion inhibitors within the microcapsules. Thin films of PS and 1% and 3% PCN were prepared for characterization purposes. A few drops were spread by a spatula on a glass slide (76 × 26 × 1 mm) and left for 24 h in the atmosphere for solvent evaporation. These techniques are discussed below.

#### 2.5.1. X-Ray Diffraction (XRD) Analysis

XRD analysis was performed using an X-ray diffractometer (Bruker, Billerica, Madison, USA). X-ray diffraction (XRD) technique was used to determine the crystallographic structure of the material. CuKα radiation was used as the anode, and the wavelength was 1.5406 Å, with a scanning rate of 3° m/s. For the raw Khulays clay, the diffraction patterns were collected at diffraction angle 2*θ* from 3 to 50 to be sure all the characteristic peaks of the constituents are included. For the other materials, the scanning range was (3–40) (2θ), the divergence slit was 0.4, and the current and voltage generators at 25 °C were 40.0 mA and 40.0 kV.

#### 2.5.2. Transmission Electron Microscopy (TEM)

The analysis was performed in the Central Laboratory at the Female Campus of King Saud University. A transmission electron microscope (JEM-1400—Joel Company, Kyoto, Japan) was used with an accelerating voltage of 100 kV. The sample preparation for TEM was as follows: a small piece of each PCN film was immersed in a suitable resin and then hardened under high temperature. Afterward, a diamond knife was used to cut thin slices of each sample to a thickness of about 70 nm. The slices were then separated by chloroform vapor. Finally, using a thin coated-carbon 200-mesh copper grid held by forceps, the samples were loaded onto the machine.

#### 2.5.3. Fourier-Transform Infrared Spectroscopy (FT-IR) Analysis

The analysis was performed using Perkin Elmer Instrument (Waltham, MA, USA). The main aim of this technique was to provide detailed information on the bond structures within compounds. It was applied to analyze the inner content of the MCs to confirm the success of loading of the corrosion inhibitors inside the MCs. The analysis was performed for all samples using the standard KBr disk method. The frequencies were in the range of 400–4400 cm^−1^.

#### 2.5.4. Scanning Electron Microscopy (SEM) and Energy-Dispersive X-Ray (EDX) Analyses

The analysis was performed in the College of Dentistry Research Center, College of Dentistry, King Saud University. The main aim of this technique was to study the shape and the average size of the microcapsules. The gold sputtering machine was used to cover the MCs with gold for imaging preparation.

The instrument used to perform SEM was JEOL Instrument model (JEC-3000FC).

#### 2.5.5. Nanoindentation Tests

Nanoindentation tests were performed on thin-film samples of PS, 1% PCN, and 3% PCN. The samples were flexible and thin having thickness of 35 ± 5 µm. To characterize the samples, tests were conducted using a depth control algorithm with a maximum depth penetration of up to 7000 nm, excluding the depth travelled during the holding period. The samples were glued to the aluminum sample holder carefully; tests were performed, and the selected maximum depth of 7000 nm was finalized to avoid the substrate effect on the sample results because the samples were very thin. The maximum recorded depth for each sample was approximately 23% of its total thickness.

### 2.6. Electrochemical Methods

In the oil industry, steel oil rigs are immersed in sea water. Steel pipes may be used to carry sea water to desalination plants. The sea environment is very corrosive to steel and steel bodies like carbon steel. Thus, we prepared a simulating solution of sea water which is 3.5% *w*/*v* NaCl or 3.5% NaCl. Thus, this study is performed in 3.5% NaCl solution. For the electrochemical study, a Potentiostat/Galvanostat instrument (Gamry Interface 3000, Warminster, PA, USA) was used. An electrochemical cell with three electrodes was used. C-steel was the working electrode; a saturated calomel electrode (SCE) was the reference electrode, and a platinum sheet was the auxiliary electrode. To evaluate the coating efficiency under different conditions, first we used the open circuit potential method for 30 min to reach a steady potential. Then, electrochemical impedance spectroscopy (EIS) was operated with an initial frequency of 10^5^ Hz to 0.5 Hz, AC voltage 10 mV/ms, and estimated Z = 100 ohm. The electrochemical impedance is generally measured by applying a small AC potential to an electrochemical cell and then measuring the current through the cell. The second method was electrochemical frequency modulation (EFM) with a base frequency 0.1 Hz and 10 mV amplitude. For the EFM, two sine waves at different frequencies were simultaneously applied to the electrochemical cell. The AC current response resulting from this perturbation consists of current components of different frequencies. Owing to the nonlinear nature of corrosion processes, responses are generated at frequencies that differ from the applied signal frequencies. Current responses can be measured at zero, harmonic, and intermodulation frequencies. In this work, the perturbation amplitude was 10 mV and frequencies of 2 and 5 Hz with a base frequency equal to 0.1 Hz were applied so that the wave form repeats after one second. All electrochemical experiments were performed in 3.5 wt.% sodium chloride NaCl solution at 20, 25, 30, and 35 °C. For evaluation of self-healing properties, the mechanical defect was performed on the coated surface with/without the inhibitor by making a cross sign (2 cm × 2 cm). EIS was followed with time, and the protection efficiency was evaluated.

## 3. Results and Discussion

### 3.1. Characterization Techniques

Various characterization techniques were applied to identify the structures and properties of these formulations. Many characterization methods such as XRD, TEM, FT-IR, SEM, and nanoindentation tests were applied. The results of these techniques are below.

#### 3.1.1. X-Ray Diffraction (XRD)

**Figure 1:**
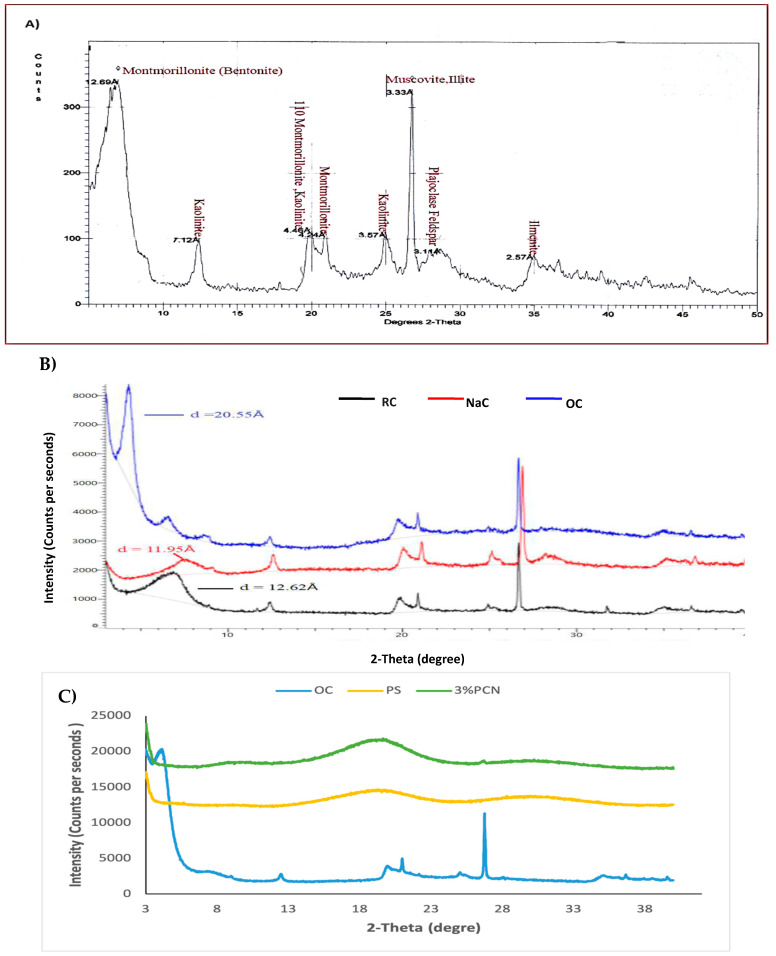
The XRD patterns of RC (**A**); RC, NaC, and OC (**B**); and OC, PS, and 3% PCN (**C**).

The XRD patterns for local Khulays raw clay (RC) is shown in Figure 1A. It shows the characteristic peaks of interlayer spacing (d-spacing) in the range of (3–50°) 2*θ*. The XRD analysis was applied to PS, NaC and the organoclay and 3% PCN to characterize the nanoscale dispersion of the clay layer in a polystyrene matrix. The XRD patterns of the prepared samples in the range of (3–40°) 2θ are shown in Figure 1B,C. The distances between the clay layers for the raw clay, NaC, OC, and PCN films were obtained from the peak position of the XRD pattern using Bragg’s equation:

2d sin*θ* = n Λ (1)

where d is the basal spacing between the clay layers, *θ* is the angle from the diffraction beam to the atomic plane, n (equal to one here) relates to the order of the reflection, and Λ is the wavelength of the X-ray radiation employed in the experiment (Λ = 1.54060 Å). These characteristic peaks are observed in the NaC and OC spectra with slight shifts due to the RC modification. The decrease between the d-spacing in the NaC compared to RC is due to the loss of water molecules [3]. However, the shifting of OC peaks to lower 2*θ* and the increase in d-spacing refer to the increase in the gap between the clay platelets. The exchange of sodium cations by CPC cations provides a good interlayer spacing [3,4,5], which means that the CPC surfactant was successfully intercalated in the clay [3,4,5].

The characteristic peaks of this Khulays clay are shown in Figure 1A. Based on a previous study on this Khulays clay [12], the dominant component is montmorillonite (35.22%). The other components are kaolinite (13.33%), mica (22.80%), quartz (8.57%), feldspar (6.66%), ilmenite (5.71), dolomite (3.81%), and gypsum (3.81%). From the XRD pattern, the starting materials RC, NaC, and OC in Figure 1B showed the normal XRD structure of the crystalline clay [12] shown in Figure 1A. The XRD patterns of OC, PS, and 3% PCN are shown in Figure 1C. We noticed that for PS, a broad diffraction peak (hump) was observed in the amorphous PS pattern. This broad peak appeared in the 3% PCN pattern with a slight increase in intensity. Meanwhile, the peak diffraction at 2*θ* = 4° in the OC pattern disappeared in the prepared 3% PCN pattern, which indicated a loss in the crystallinity of the prepared PCN. This disappearance results from the insertion of PS chains between the nanolayers of the OC. Moreover, we can deduce the possibility of an exfoliated structure in the prepared 3% PCN as found by other studies [5,14]. The XRD analysis can be used with the TEM technique to confirm the presence of an exfoliated structure. The TEM analysis was performed to confirm these results.

#### 3.1.2. Transmission Electron Microscopy (TEM)

**Figure 2:**
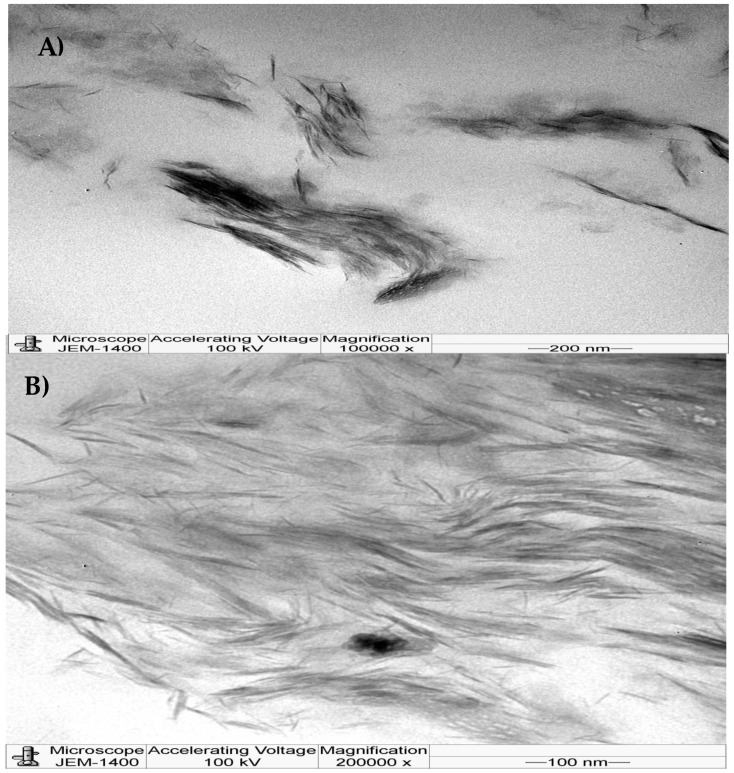
TEM images of 3% PCN at two magnifications (**A**,**B**).

TEM was performed to determine the internal structure of the PCN at the nanoscale level. In Figure 2, the TEM images of 3% PCN are presented at high magnifications (A and B). As shown, the dark area (hair-like) refers to clay, whereas the light area refers to polystyrene. We can clearly observe the clay nanolayers dispersed throughout the polymer matrix, which indicates the strong possibility of an exfoliated structure of 3% PCN formulation as can be observed at the 200,000 magnification. Thus, combining the XRD and TEM results, it can be concluded that 3% PCN has an exfoliated structure as shown in similar studies [16,17,18].

#### 3.1.3. Fourier-Transform Infrared Spectroscopy (FT-IR)

##### FT-IR Spectra of RC, NaC, OC, and CPC

**Figure 3:**
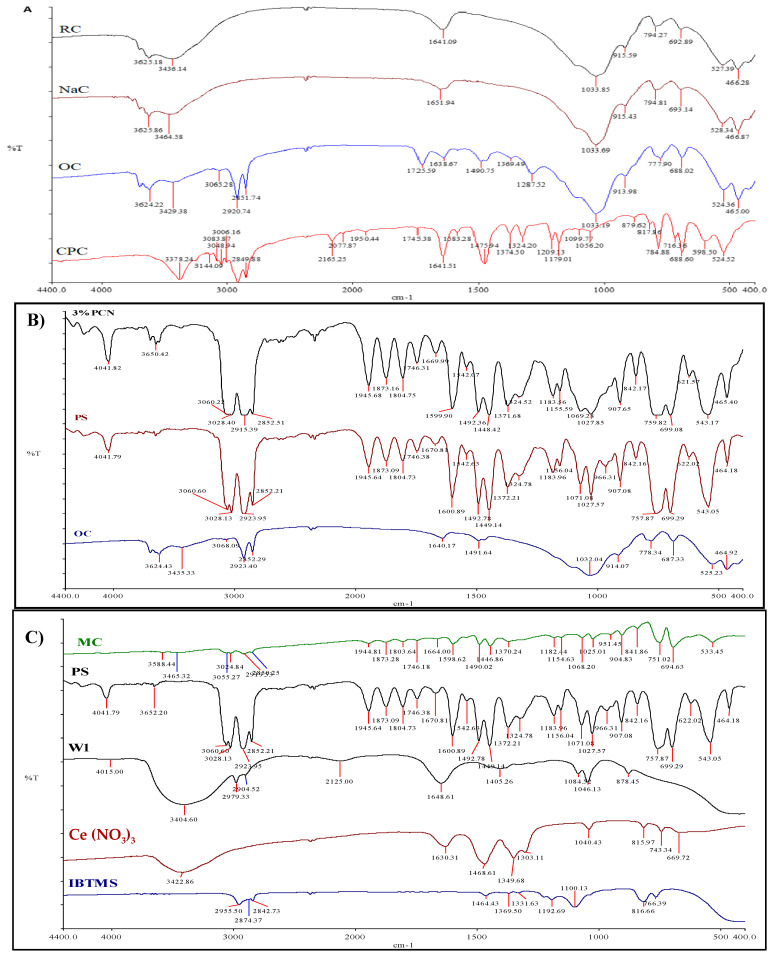
FT-IR spectra of RC, NaC, OC, and CPC (**A**); FTIR of OC, PS, and 3% PCN (**B**); and FT-IR of MC, PS, W1 IBTMS, and Ce(NO_3_)_3_ (**C**).

The full FT-IR spectra of the RC, NaC, OC, and CPC samples are illustrated in Figure 3A. For RC, the bands at 3696 cm^−1^ and 3625 cm^−1^ are due to the -OH band stretching for Si-OH and Al-OH, respectively. The broad band at 3428 cm^−1^ corresponds to the stretching vibrations of the hydroxyl groups, and the absorption band of -OH bending is shown at 1637 cm^−1^ [4,5]. The strong absorption band at 1032 cm^−1^ is ascribed to Si-O stretching vibrations of layered silicates. The bands at 528 and 466 cm^−1^ are related to the Al-O-Si bending and Si-O-Si bending vibrations [4,5,17,18,19]. The absorption bands for NaC are 3627 cm^−1^ (-OH stretching vibration of (Al-OH) and 1033 cm^−1^ (Si-O stretching vibrations). The absorption bands of NaC found at 3432, 109, and 1632 cm^−1^ are characteristic of the stretching and bending vibrations of the interlayer water of layered silicates [14,17,18,19]. The bending-in-plane vibrations of the –OH groups in NaC at 1632 cm^−1^ are shifted to 1636 cm^−1^ in the FT-IR of the OC sample. The broad band observed in NaC at 3432 cm^−1^ corresponding to the stretching vibrations of OH (interlayer water) is shifted to 3428 cm^−1^ in OC, due to the intercalation of the surfactant molecules. A pair of strong peaks at 2915 and 2849 cm^−1^ can be assigned to the asymmetric and symmetric stretching vibrations of the C-H in the alkyl chains of the CPC molecules [3,4,12,19]. These sharp peaks are shifted to 2929 and 2852 cm^−1^, indicating the intermolecular attractions between adjacent alkyl chains of CPC in NaC galleries [4,12,19]. In addition, a new weak band in the OC spectrum at 1195 cm^−1^ may refer to the (C-N) bond stretching from CPC, with a little shift to a lower frequency than the present one in the CPC spectrum [5,14,17]. The FTIR spectrum of OC differs from the RC and NaC clay spectra with the presence of additional absorption bands, which proves the success of CPC cation intercalation between the silicate layers (d_001_ spacing), since the absorption band at 3065 cm^−1^ is related to the stretching vibrations of sp2 (C–H) bonds from CPC with its bending at 1493 cm^−1^_._

##### FT-IR Spectra of OC, PS, and 3% PCN

The FT-IR spectra of OC, PS, and 3% PCN are shown in Figure 3B. Regarding the OC pattern, the absorption of the stretching vibration of (O-H) bonds in Al-OH appears at a frequency of 3624 cm^−1^ with a bending vibration at 914 cm^−1^. The strong absorption at 1032 cm^−1^ corresponded to the stretching vibration of the (Si-O) bond. The two absorption peaks observed at 525 and 464 cm^−1^ were related to the (Al-O-Si) and (Si-O-Si) stretching vibrations. The strong and sharp absorption bands at 2923 and 2852 cm^−1^ are ascribed to the asymmetric and symmetric stretching vibrations of the C-H in the alkyl chains of the surfactant CPC molecules, as shown in previous studies [5,14]. In the PS spectrum, the two absorption bands at 3060 and 3028 cm^−1^ represent the aromatic stretching vibrations of sp^2^ (C-H). Asymmetric and symmetric vibrations of aliphatic (C-H) were observed as a strong absorption band at 2923 and 2852 cm^−1^ with its bending vibration at 1449 cm^−1^. The absorption peaks observed at 1071, 1027, and 966 cm^−1^ are ascribed to the in-plane bending of (C-H), while the absorption peaks observed at 907, 757, and 699 cm^−1^ are attributed to the out-of-plane bending of (C-H) as reported by Krimm et al. [19]. Comparing the IR spectra of both PS and OC, it is noticeable that the presence of the characteristic absorption bands of PS in the prepared polystyrene clay nanocomposites of 3% PCN occur by inserting polystyrene chains between the organically modified clay layers (interlayer). Based on the above discussion, we successively prepared a polystyrene/organoclay nanocomposite.

##### FT-IR of IBTMS, Ce(NO_3_)_3_, W_1_, and MC

Polystyrene microcapsules (MCs) loaded with the corrosion inhibitors cerium and silane were prepared according to the method described by Cotting et al. [15]. To confirm the encapsulation processes, the FT-IR spectra of Ce(NO_3_)_3_·6H_2_O, IBTMS, W1, and MCs are shown in Figure 3C. In the IBTMS spectra, the absorption band at 2842 cm^−1^ represents (C-H) stretching of the methoxy group. The two sharp absorption bands at 2955 and 2873 cm^−1^ are attributed to the asymmetric and symmetric stretching vibrations of the (C-H) bond in (-CH_2_-) and its deformation vibration band at 1464 cm^−1^ [19]. A broad, high-intensity absorption band is present at 1100 cm^−1^, which may reflect the stretching vibrations of (Si-O-Si) in the silane [15,19,20]. The Si-O-C stretching vibrations were detected at 816 cm^−1^ as an extreme band in the spectrum [19]. For the Ce(NO_3_)_3_·6H_2_O spectrum, the broad peak at 3422 cm^−1^ represents OH stretching vibration and its bending at 1630 cm^−1^ [21]. The absorption bands at 1468 and 1349 cm^−1^ correspond to the asymmetric and symmetric stretching vibrations in the (N-O) bond [20,22]. The peak observed at 1040 cm^−1^ corresponds to the stretching vibration of free (NO_3_^−^) ions and their bending vibration at 815 cm^−1^ [23]. The disappearance of the stretching vibrations of the methoxy group from the silane (C-H) indicates that the methoxy groups were hydrolyzed completely during W_1_ preparation. In the range of 1455–1376 cm^−1^ and 1046 cm^−1^, the typical vibrations of nitrates can be observed but with a much weaker intensity. Furthermore, a new intense band at 878 cm^−1^ was observed. A possible explanation for this new band is the formation of bonds between the cerium and silicon atoms in silanol that result in new stretching vibrations between them. Taking into consideration the spectra discussed above and the studied vibrations of PS, it is evident that the FT-IR spectrum of the prepared microcapsules MCs indicates the success of the encapsulation of the corrosion inhibitor solution W1 by PS.

#### 3.1.4. Scanning Electron Microscopy (SEM)

**Figure 4:**
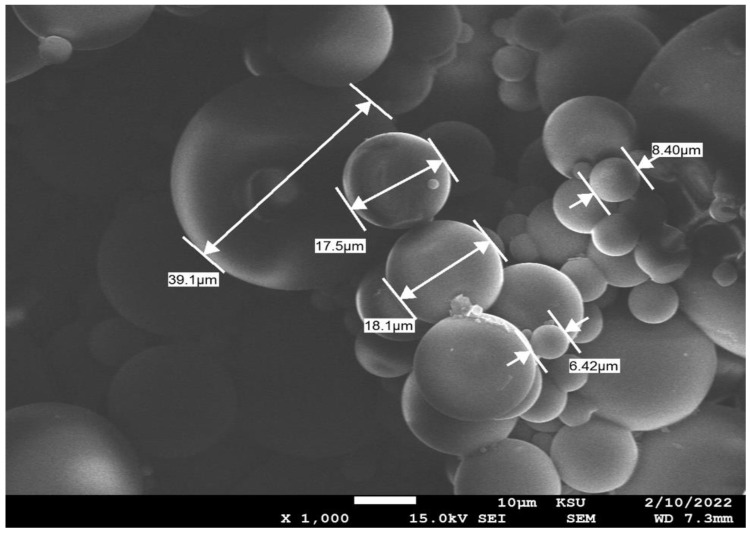

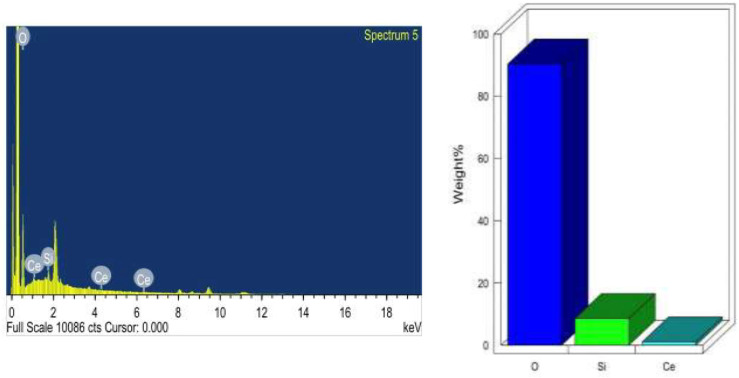
SEM images showing the diameter of MCs (top photo) and EDX analysis of MCs (bottom photo).

**Figure 5:**
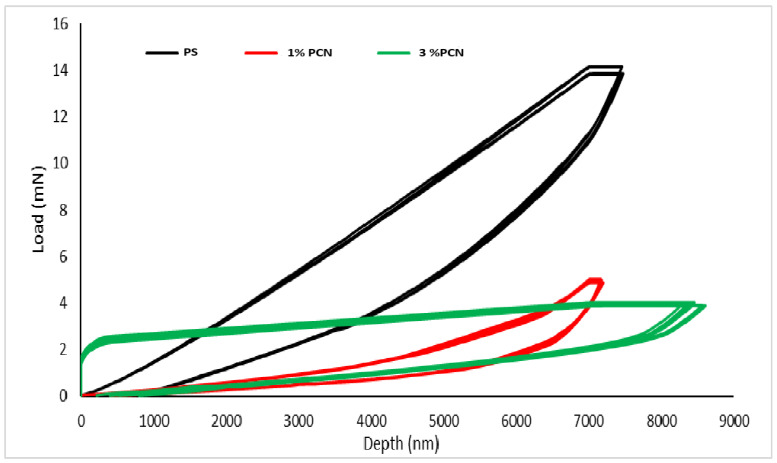
Load vs. depth curves for thin-film samples.

It can be observed from Figure 4 that we obtained spherical capsules with a diameter that varied in the range of 6–39 µm. Furthermore, a mononuclear structure with a nonporous surface was observed, which coincides with the findings of Cotting et al. [15]. Moreover, SEM-EDX proved the presence of Ce^+3^ and Si^+4^ inside these microcapsules as shown in Figure 5, with the percentage of silicone exceeding that of cerium, which proved the successful encapsulation of Ce(NO_3_)_3_ and silane in these microcapsules.

#### 3.1.5. Nanoindentation

Figure 5 shows the results of the obtained load versus depth curves for all samples. It can be seen in this figure that the highest load was recorded for the PS sample, indicating the strength of the PS film. On the other hand, the PCN films (1% and 3%) showed poor resistance to the load under the same testing conditions. In the case of 1% PCN, a sudden penetration at lower loads can be observed, which suggests that the surface of this film is flexible and easily penetratable [24]. In the case of 3% PCN, the sudden ramp in load is observed, which is directed toward a relatively hard film surface in comparison to 1% PCN, followed by prolonged depth penetration. As shown in Figure 5, 3% PCN film initially resisted the applied load on its surface; then, after a certain limit, the indenter abruptly penetrated inside the film suggesting a rupture at the film’s surface and poor resistance to load.

Table 1 shows the hardness and reduced modulus values (MPa) obtained from the sample analysis. The reduced modulus is the modulus representing the elastic deformation both in the sample and at the tip of the diamond indenter. A significant reduction in both the hardness and reduced modulus (MPa) can be observed for the PCN film in comparison to the PS film. From the obtained results, it can be concluded that PCN films are not as effective in terms of hardness and reduced modulus as PS and they are underperformed in load-bearing resistance and possess less resistance to penetration compared to PS. As can be noticed from Table 1, 3% PCN has a slightly higher hardness than 1% PCN. Although these results are in air and not in the corrosive medium, they indicate that the penetration of corrosive materials is slightly harder for 3% PCN. On the other hand, the reduced modulus of 1% PCN is about 2.9 that of 3% PCN.

In a previous study of our research group [12], we used Khulays organoclay to prepare polystyrene clay nanocomposite and differential scanning calorimetry (DSC) was applied to 1–10% PCN. The glass transition temperature decreased by 1–3% PCN compared to PS. Thus, the polymer film became softer with increasing clay loading from 1 to 3% [12]. Moreover, the adhesion test was performed on the dry samples using the crosscut (cross-hatch) testing for coatings of PS and 1–10% PCN on CS [13]. Our results show that for the PS and 1% and 5% PCN coatings, the area removed was 35–65%. However, for 3% PCN, 0% of the coating area was removed. Thus, 3% PCN provides the best coating adhesion. This improvement in adhesion indicates that the prepared 3% PCN filled the voids and crevices on C-steel surface. Considering the nanoindentation results and our previous findings about adhesion tests, only 3% PCN will be used in the remainder of this study to test the smart coating properties.

### 3.2. Electrochemical Methods

In this study, C-steel rods coated with PS, PCN, and PCN(MC) were used. All electrochemical experiments were performed after immersing the coated C-steel in a 3.5 wt.% NaCl solution. The electrical impedance of a circuit represents its ability to resist the current flow as it passes through it. The effects of temperature, time, and mechanical damage are illustrated below.

#### 3.2.1. Electrochemical Impedance Spectroscopy (EIS) as a Function of Temperature

**Figure 6:**
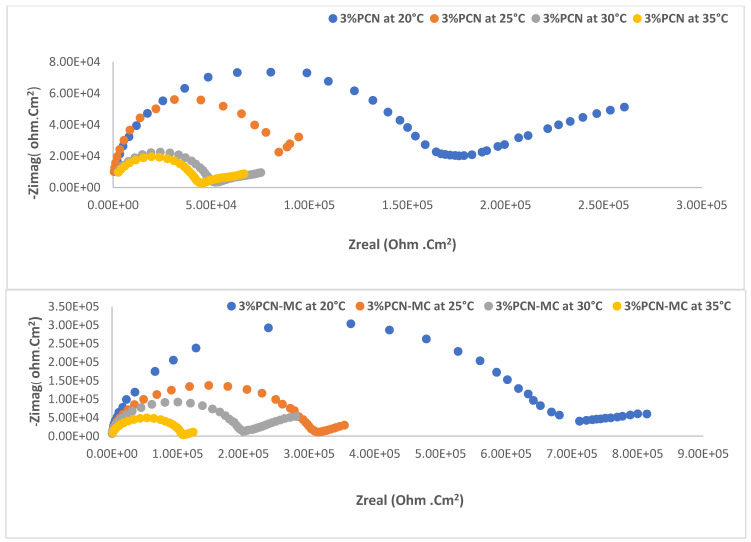
The Nyquist plot of 3% PCN (**top photo**) and 3% PCN(MC) in 3.5% NaCl at different temperatures (**bottom photo**).

**Figure 7:**
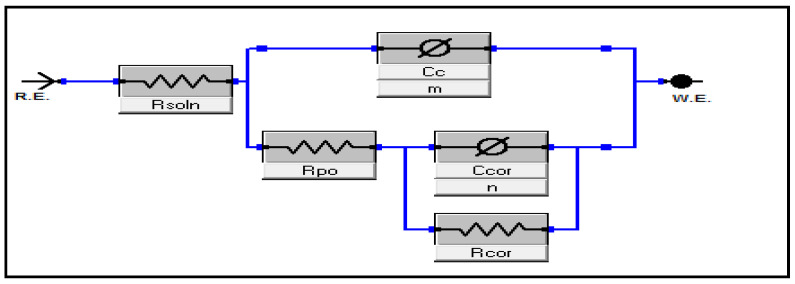
Schematic diagram of the coated C-steel-equivalent circuit.

All chemical reactions are influenced by temperature changes, which affect the corrosion rates of the metal and alloy [2,25,26]. In this work, we studied the effect of the change in temperature on the corrosion behavior of C-steel coated with PS, 3% PCN, and 3% PCN(MC) in 3.5 wt.% NaCl. The Nyquist plots of the tested samples are shown in Figure 6 for 3% PCN and 3% PCN(MC). Two semi-circles can be observed in both coated systems. The first one in the high-frequency portion is related to the coating system’s resistance to the electrolyte passage (R_Po_) and capacitance (Cc). The second semi-circle in the lower frequency portion is ascribed to the electrochemical reaction of the metal surface and the double-layer capacitance [2,24,25]. The decreasing semi-circle diameter with increasing temperature up to 35 °C resulted from the progressive diffusion and increasing attack of the corrosive agents which increased with higher temperature as was documented by others [2,25,26]. The electrical circuit in Figure 7 comprises the electrolyte resistance (R_soln_) and a constant phase element representing the coating capacitance (Cc). This coating capacitance is in parallel to the coating resistance to the passage of electrolytes (R_Po_), the constant phase elements representing the double-layer capacitance between the metal surface/electrolyte solution (C_Cor_) and the charge transfer resistance across the metal surface (R_cor_). As reported in the literature for the coated substrates [11,13,25], the first semi-circle in the high-frequency region was related to the resistance and capacitance of the protective coating and its properties. The second semi-circle in the low-frequency region was attributed to the electrochemical reactions on the C-steel surface. Illustrations of equivalent circuits are shown in Figure 7. These experimental results were fitted via the Gamry software (Interface 3000).

The impedance parameter data for all coated samples are listed in Table 2. These data were extracted by fitting the Nyquist plots of the coated samples to an equivalent circuit, which depicts the experimental results extracted from Gamry software. The electrical circuit consists of the electrolyte resistance (R_Sol_), the constant phase element representing the coating capacitance (C_c_), the coating resistance to the passage of electrolytes (R_Po_), the constant phase elements representing the double-layer capacitance between the metal surface/electrolyte solution (C_Corr_), and the charge transfer resistance across the metal surface (R_Corr_). It is shown in Table 2 that the increase in the value of corrosion resistance (R_Corr_) is coupled with a decrease in the corrosion capacitance C_Corr_ value at all temperatures compared to PS.

Thus, the enhancement of the anticorrosive coating efficiency means that the inhibitor layer growth over the metal’s surface could limit water access to the metal’s surface [23]. In fact, water has a relatively high dielectric constant; thus, when inhibitors replace water molecules, there is a reduction in capacitance value.

Table 2 shows that the corrosion resistance values R_Cor_ and R_po_ of the PS, 3% PCN, and 3% PCN(MC) samples decreased when the temperature was increased from 20 to 35 °C. The same trend was observed for the R_po_ values. The C_cor_ and C_c_ values increased as the temperature increased. Based on the R_corr_ values, intercalating a small amount of the organoclay in the PS matrix increased the coating resistance compared to the pure PS samples.

This is the result of the clay nanolayers lengthening the corrosive ion diffusion pathways. For PCN, the protection efficiency increased from 73.58% at 20 °C to 62.17% when the temperature was 35 °C. On the other hand, the PCN(MC) coating impregnated with corrosion inhibitors accomplished greater protection against the same temperature changes. The 3% PCN(MC) protection efficiency (PE%) was 99.70% at 20 °C and decreased only to 79.21% at 35 °C as calculated from Equation (2).

(2) $$\%PE=(1-\frac{\left(Rcorr\right)blank}{\left(Rcorr\right)inhib.})\times 100$$

This response at higher temperatures demonstrates the smart behavior of this PCN impregnated with inhibitors. Thus, the improved protection effectiveness of the 3% PCN(MC) coating sample is likely to be a combination of several factors. Among these is the exfoliated structure of PCN. Furthermore, microcapsules loaded with (Ce^+3^) and silanol enhanced the coating performance.

#### 3.2.2. Electrochemical Frequency Modulation (EFM) as a Function of Temperature

According to the obtained corrosion parameters, the corrosion rate (CR) and corrosion current density (I_Corr_) are listed in Table 3. Based on the experimental casualty values of our data, they are close to the theoretical values, that is, (2.00) and (3.00), respectively, which provide an internal check of the validity of the EFM measurement.

Based on the corrosion parameters obtained from the EFM spectra, we noticed that the corrosion current density values (I_corr_) increased from 4.87 × 10^−2^ to 1.38 × 10^−1^ µA/cm^2^ when the temperature increased from 20 to 35 °C in the PS samples. This indicates a reduction in corrosion resistance when the environmental temperature increases. Moreover, the corrosion current density value (I_corr_) was 3.49 × 10^−3^ µA/cm^2^ for the 3% PCN sample at 20 °C, whereas at 35 °C, the current density reached 5.54 × 10^−2^ µA/cm^2^. In this study, MCs loaded with corrosion inhibitors such as cerium ions are impregnated in the coating layers to further improve the inhibition efficiency of PCN. Based on the current density data obtained from the EFM plots of 3% PCN(MC), we noticed the I_corr_ values at (20–35 °C) increased to (3.49 × 10^−3^–5.54 × 10^−2^ µA/cm^2^), compared to PS and 3% PCN. The CR values of the PS, 3% PCN, and 3% PCN(MC) are (4.69 × 10^−3^, 6.93 × 10^−4^, 3.36 × 10^−4^) mpy at 20 °C. Thus, the corrosion rate (CR) of the coated samples improved compared to PS and the presence of the inhibitors further improved the coating’s properties.

The protection efficiency percentages (%PEs) were estimated from the equation:

(3) $$\%PE=\frac{\left(icorr\right)blank-\left(icorr\right)inhib}{\left(icorr\right)blank}\times 100$$

Furthermore, the incorporation of organoclay into the PS matrix enhanced the inhibition protection as the %PE for 3% PCN is estimated to be 85.21% at 20 °C and 89.44% with 35 °C. In addition, the protection efficiency of 3% PCN(MC) increased by 92.83% at 20 °C and by 96.15% even when the environmental temperature was increased to 35 °C. The presence of the organoclay slowed the corrosion rate. Moreover, the corrosion rate decreased in the presence of cerium ions and silanol.

#### 3.2.3. Evaluation of Self-Healing Properties

The corrosion process is initiated when the aggressive chloride ions reach the substrate. It is worth mentioning that the chemical interaction of the coating with an inhibitor is difficult to predict as it depends on the coating composition and properties. In our study, corrosion inhibitors such as cerium ions and silanol were encapsulated in polystyrene microcapsules. Numerous previous studies have used the electrochemical impedance spectroscopy (EIS) technique to study the self-healing behavior of protective coatings to confirm the role of MCs on the prepared nanocomposite coating [27,28]. Thus, in the present study, EIS measurements were set up for 3% PCN and 3% PCN(MC) coatings. The measurements were performed during exposure to a corrosive electrolyte 3.5 wt.% NaCl at a room temperature of 20 °C at different immersion times. We observed an increase in the corrosion resistance values R_Cor_ in Table 4. The corrosion resistant (R_corr_) values of 3% PCN and 3% PCN(MC) after 2 h of immersion equaled (3.85 × 10^6^ and 6.08 × 10^6^ Ω·cm^2^), and they increased to 3.66 × 10^7^ and 7.22 × 10^9^ Ω·cm^2^ after 24 h. Thus, the impregnated PCN with MCs improved the protection efficiency by approximately 99% as shown in Table 4. These improvements in resistance were due to the release of the inhibitors from the microcapsules as shown by Cotting et al. and Danaee et al. [15,28]. It was hard to compare our smart system formulation with others due to the difference in the matrix and thickness and the coupling agent. However, we found that like Cotting et al. [15], after 4 h of immersion, the R_corr_ values for the undoped system changed from 31.1 to 121.3 k Ω·cm^2^ using epoxy coating. In our study, the R_corr_ values for the undoped system changed from 1.04 × 10^6^ to 1.63 × 10^6^ Ω·cm^2^, which reflect the enhanced property of our smart system.

In silane-based coatings, the addition of cerium ions has been reported by some authors as having a beneficial result, owing to enhanced cross-linking [15]. To evaluate the self-healing mechanism of the inhibitors, we studied the effect of these inhibitors after performing a mechanical defect. EIS was followed with time for 30 h, as presented in Table 5.

Comparing the R_corr_ without defect with R_corr_ values after the mechanical defect, we noticed the decrease in the corrosion resistance after the mechanical defect at each time interval for both systems as the metal surface is exposed partially to the corrosive medium. For 3% PCN samples with a mechanical defect, the R_corr_ values decreased with immersion times. This collapse in the coating system was due to the scratched area; hence, the corrosive ions freely reached the metal surface without any interruption. However, after 24 and 30 h, we noticed a slight increase in the corrosion resistance. This behavior could result from the formation of an oxide metal film on the C-steel surface for 3% PCN. Thus, the metal film oxide acts as a barrier against corrosive ions.

Furthermore, the corrosion resistant (R_corr_) values of 3% PCN and 3% PCN(MC) after 2 h of immersion increased from 1.59 × 10^3^ Ω·cm^2^ and 2.94. Ω·cm^2^ to 7.32 × 10^3^ and 2.75 × 10^6^ Ω·cm^2^ after 24 h. The impregnated MCs into the coating systems improved the protection efficiency by 99.5% compared to 3% PCN with a defect. These improvements in resistance are due to the release of inhibitors from the microcapsules, which healed the surface. It is worth mentioning that although 3% PCN(MC) has a higher R_corr_ at each time interval, it reached its highest R_corr_ after 2 h then decreased gradually with time. These results are consistent with the results of previous studies on the self-healing performance in protective coatings [15,27,28]. Regarding the corrosion resistance values (R_corr_) of the 3% PCN(MC), we noticed almost a thousand times increase in resistance after six hours of immersion and a jump in the corrosion resistance after 24 h of immersion. This increase continued until 30 h of immersion, and the corrosion resistance reached 1.60 × 10^8^ Ω·cm^2^ for 3% PCN(MC). This indicates that the corrosion inhibitors were released from the microcapsule and provided higher protection.

It is believed that an electrochemical reaction will occur when corrosive ions diffuse through the coating layer to the C-steel surface. Anodic oxidation on the C-steel surface releases free electrons and forms iron cations via the following reactions:

(4) $${2Fe}_{\left(S\right)}⇌{2Fe}_{\left(aq\right)}^{2+}+{4e}^{-}$$

In the presence of oxygen and water, an unstable ferrous hydroxide product ${Fe(OH)}_{2}$ will be formed. Then, it will be oxidized to form a reddish-brown product, which is ferric hydroxide $Fe{\left(OH\right)}_{3}$. Thus, it would provide a thin protective layer. The diffused corrosive ions would trigger the release of the inhibitors from our prepared MCs. Self-healing phenomena may occur in two steps. In the first step, the silanol groups (Si-OH) are adsorbed on the C-steel surface by hydroxide groups with a hydrogen bond. Then, the adsorbed silanol group condenses with metal hydroxide on the C-steel surface to form an (M–O–Si) and this would provide a hydrolysable film on the electrode surface. The condensation of silanol may occur, followed by hydrogen bonding with the hydroxyl groups present on the surface of the C-steel, which is accompanied by a decrease in the corrosion reaction by adsorption as well as an increase in the coating adhesion as suggested by others [29,30]. Furthermore, an insoluble oxide/hydroxide cerium film may be formed and inhibit the cathodic reaction [31,32,33,34].

This film may be formed according to the following reaction:

(5) $$2H_{2}O+\;O_{2}+4\;e^{-}\to \;\;4{OH}^{-}$$

(6) $$\;\;{Ce}^{+3}+2{OH}^{-}\;\to \;\;{Ce(OH)}_{2}^{2+}+e^{-}$$

(7) $${Ce(OH)}_{2}^{2+}+2{OH}^{-}\;\to {CeO}_{2}+2H_{2}O$$

In conclusion, the self-healing mechanism consists of two processes. The first one is the silanol functionality, which relies on its coupling agent property and the formation of a non-soluble adsorbed film on the surface of the C-steel electrode. The second one is the reduction in cerium.

## 4. Conclusions

1. We prepared a polymer clay nanocomposite coating using modified Khulays clay. The modification process of our coating was followed by many characterization processes like FT-IR, XRD, TEM, SEM-EDX, and the nanoindentation technique. In addition, we applied several electrochemical techniques. Polystyrene microcapsules loaded with corrosion inhibitors were successively prepared by double emulsion solvent evaporation methods. To prepare smart coatings, we prepared 3% PCN coatings then doped them with corrosion inhibitors cerium and silane to increase the coating efficiency.
2. The protective coating efficiency was evaluated under temperature effect using several electrochemical techniques such as EIS, EFM, and LPR in 3.5% NaCl solutions. The results show that 3% PCN impregnated with MCs showed enhanced protection efficiency reaching over 99% compared to the non-impregnated 3% PCN coating formulation.
3. The self-healing performance of PCN(MC) coatings was supported by related experiments, such as scratch test and temperature effect. The self-healing mechanism related to the diffusion mechanism of the inhibitors released from the microcapsules consists of two processes. The first one is the silanol functionality, which relies on its coupling agent property and the formation of a non-soluble adsorbed film on the surface of the C-steel electrode. The second one is the reduction in cerium. The results of our coating system are unique and provide promising applications.
4. The prepared smart coatings showed superior corrosion protection as shown by the electrochemical measurements that could provide great potential for many applications in the coating of steel and carbon steel in the oil industry.

## Figures and Tables

**Table 1:** Hardness and modulus values are presented below.

Sample Code	Hardness (MPa)	Reduced Modulus (MPa)
PS	24.19	143.47
1% PCN	5.77	85.07
3% PCN	6.68	29.35

**Table 2:** EIS-fitted data of C-steel coated with PS, 1% PCN, 3% PCN, and 3% PCN(MC) after 1 h of immersion in 3.5 wt.% NaCl at different temperatures.

Sample	T (°C)	R_Cor_ (Ω·cm^2^)	R_PO_ (Ω·cm^2^)	C_Cor_ (F·cm^−2^)	Cc (F·cm^−2^)	%PE
PS	20	2.38 × 10^5^	1.03 × 10^5^	1.07 × 10^−5^	1.88 × 10^−10^	-
	25	5.55 × 10^4^	1.37 × 10^5^	1.45 × 10^7^	1.85 × 10^−10^	-
	30	3.09 × 10^4^	3.09 × 10^4^	7.46 × 10^2^	1.20 × 10^−5^	-
	35	2.64 × 10^4^	4.95 × 10^2^	4.04 × 10^−5^	6.66 × 10^−7^	-
3% PCN	20	9.01 × 10^5^	1.41 × 10^5^	3.42 × 10^−6^	1.64 × 10^−10^	79.91
	25	2.18 × 10^5^	6.51 × 10^4^	6.31 × 10^−6^	1.76 × 10^−10^	74.50
	30	1.21 × 10^5^	4.52 × 10^4^	1.86 × 10^−5^	2.24 × 10^−10^	74.46
	35	6.98 × 10^4^	4.00 × 10^4^	1.66 × 10^−5^	2.31 × 10^−10^	62.17
3% PCN(MC)	20	6.09 × 10^7^	4.92 × 10^5^	2.79 × 10^−6^	2.29 × 10^−10^	99.70
	25	3.21 × 10^6^	2.84 × 10^5^	1.11 × 10^−5^	2.44 × 10^−10^	98.27
	30	7.68 × 10^5^	1.87 × 10^5^	5.98 × 10^−6^	2.89 × 10^−10^	95.98
	35	1.27 × 10^5^	1.05 × 10^5^	2.77 × 10^−5^	3.06 × 10^−10^	79.21

**Table 3:** EFM data of PS, 3% PCN, and 3% PCN(MC) in 3.5% NaCl at different temperatures.

Sample	T (°C)	I_corr_ (µA/cm^2^)	Corrosion Rate (mpy)	%PE
PS	20	2.79 × 10^−2^	2.69 × 10^−3^	-
	25	3.24 × 10^−1^	3.11 × 10^−2^	-
	30	1.28	1.23 × 10^−1^	-
	35	1.44	1.38 × 10^−1^	-
3% PCN	20	7.20 × 10^−3^	6.93 × 10^−4^	85.21
	25	1.75 × 10^−2^	1.68 × 10^−3^	94.50
	30	1.39 × 10^−1^	1.34 × 10^−2^	89.14
	35	1.52 × 10^−1^	1.47 × 10^−2^	89.44
3% PCN(MC)	20	3.49 × 10^−3^	3.36 × 10^−4^	92.83
	25	7.38 × 10^−3^	7.10 × 10^−4^	97.70
	30	2.33 × 10^−2^	2.24 × 10^−3^	98.18
	35	5.54 × 10^−2^	5.33 × 10^−3^	96.15

**Table 4:** EIS-fitted data with time for 3% PCN and 3% PCN(MC) in 3.5% NaCl at 20 °C without the mechanical defect.

Sample	Time (Hrs.)	R_Cor_ (Ω·cm^2^)	R_PO_ (Ω·cm^2^)	C_Cor_ (F·cm^−2^)	C_C_ (F·cm^−2^)	%PE
3% PCN	2	3.85 × 10^6^	2.38 × 10^6^	8.81 × 10^−8^	1.97 × 10^−10^	-
	4	1.04 × 10^6^	5.53 × 10^5^	1.34 × 10^−6^	1.90 × 10^−10^	-
	6	1.50 × 10^6^	4.83 × 10^3^	3.90 × 10^−6^	1.88 × 10^−10^	-
	24	3.66 × 10^7^	1.32 × 10^5^	2.31 × 10^−6^	1.95 × 10^−10^	-
3% PCN(MC)	2	6.08 × 10^6^	1.11 × 10^6^	1.99 × 10^−7^	1.80 × 10^−10^	36.67
	4	1.63 × 10^6^	6.45 × 10^5^	3.65 × 10^−7^	1.94 × 10^−10^	36.19
	6	1.97 × 10^6^	4.44 × 10^5^	4.90 × 10^−7^	1.94 × 10^−10^	23.85
	24	7.22 × 10^9^	6.02 × 10^6^	1.49 × 10^−7^	1.77 × 10^−10^	99.02

**Table 5:** EIS-fitted data with time for 3% PCN and 3% PCN(MC) in 3.5% NaCl at 20 °C with the mechanical defect.

Sample	Time (Hrs.)	R_Cor_ (Ω·cm^2^)	R_PO_ (Ω·cm^2^)	C_Cor_ (F·cm^−2^)	C_C_ (F·cm^−2^)	%PE
3% PCN mechanical defect	2	1.59 × 10^3^	1.02 × 10^2^	3.41 × 10^−4^	5.36 × 10^−4^	-
	4	5.19 × 10^2^	23.83	8.80 × 10^−4^	6.81 × 10^−4^	-
	6	3.87 × 10^2^	19.46	1.02 × 10^−3^	8.73 × 10^−4^	-
	24	7.32 × 10^3^	17.61	9.10 × 10^−4^	5.96 × 10^−4^	-
	30	6.39 × 10^5^	10.48	1.44 × 10^−3^	4.92 × 10^−4^	-
3% PCN(MC) mechanical defect	2	2.94 × 10^3^	208.4	1.27 × 10^−4^	8.60 × 10^−5^	45.80
	4	1.57 × 10^3^	89.89	2.82 × 10^−4^	2.02 × 10^−4^	66.87
	6	1.36 × 10^3^	60.1	3.48 × 10^−4^	6.52 × 10^−4^	71.57
	24	2.75 × 10^6^	14.28	2.63 × 10^−3^	2.17 × 10^−3^	99.73
	30	1.60 × 10^8^	13.76	2.62 × 10^−3^	2.42 × 10^−3^	99.996

## Data Availability

The original contributions presented in the study are included in the article.
